# CD147 Is a Promising Target of Tumor Progression and a Prognostic Biomarker

**DOI:** 10.3390/cancers11111803

**Published:** 2019-11-16

**Authors:** Alexandra Landras, Coralie Reger de Moura, Fanelie Jouenne, Celeste Lebbe, Suzanne Menashi, Samia Mourah

**Affiliations:** 1 INSERM UMRS 976, Team 1, Human Immunology Pathophysiology & Immunotherapy (HIPI), University of Paris, 75010 Paris, France; alexandra.land@hotmail.fr (A.L.); coralie.reger@aphp.fr (C.R.d.M.); fanelie.jouenne@aphp.fr (F.J.); celeste.lebbe@aphp.fr (C.L.); suzanne.menashi@gmail.com (S.M.); 2 Pharmacogenomics Department, Assistance Publique-Hôpitaux de Paris (AP-HP), Saint Louis Hospital, 75010 Paris, France; 3 Dermatology Department and Centre d’Investigation Clinique (CIC), Assistance Publique-Hôpitaux de Paris (AP-HP), Saint Louis Hospital, 75010 Paris, France

**Keywords:** CD147, biomarker, tumor microenvironment, prognosis, targeted therapy

## Abstract

Microenvironment plays a crucial role in tumor development and progression. Cancer cells modulate the tumor microenvironment, which also contribute to resistance to therapy. Identifying biomarkers involved in tumorigenesis and cancer progression represents a great challenge for cancer diagnosis and therapeutic strategy development. CD147 is a glycoprotein involved in the regulation of the tumor microenvironment and cancer progression by several mechanisms—in particular, by the control of glycolysis and also by its well-known ability to induce proteinases leading to matrix degradation, tumor cell invasion, metastasis and angiogenesis. Accumulating evidence has demonstrated the role of CD147 expression in tumor progression and prognosis, suggesting it as a relevant tumor biomarker for cancer diagnosis and prognosis, as well as validating its potential as a promising therapeutic target in cancers.

## 1. Introduction

Tumor complexity represents a challenge with regard to the development of new therapeutic strategies [1,2]. Tumor development is a dynamic process involving cooperation between different cellular and noncellular elements of the tumor microenvironment (TM) such as fibroblasts, endothelial cells, pericytes and immune cells including lymphocytes B and T, tumor-associated macrophages, natural killers, as well as extracellular components (growth factors, hormones, cytokines) surrounded by a blood/lymphatic vascular network present in a complex extracellular matrix (ECM). Recently, cancer progression and drug resistance have been proposed to result from the interaction of the tumor cells with their microenvironment [3,4]. Targeting molecules capable of modulating the TM in favor of the tumor cell can lead to efficient therapeutic strategies. In addition, the microenvironment, through the proteolytic degradation of its ECM, can further promote tumor progression and ease the metastasis process [5]. Matrix metalloproteinase (MMPs), which are the main ECM degrading enzymes, are often overexpressed in cancer and are associated with a poor prognosis [6]. Understanding the regulation of their production and activation appears to be important for the development of new therapeutic strategies.

CD147 (cluster of differentiation 147) is a glycoprotein initially known as a regulator of MMPs, through cell–matrix and cell–cell interaction and represents a potential target for cancer therapy [7]. CD147 was found to be overexpressed in cancer cells and is believed to promote their malignant proprieties, such as proliferation and inhibition of cancer cell apoptosis [8]. During the past decades, CD147 was implicated, in addition to its role as a regulator of MMPs, in several other functions due to its ability to regulate or to bind with different molecular partners and hence has the ability to modulate several cellular pathways [9]. In particular, through its association with certain monocarboxylate transporter (MCTs), CD147 was shown to act as a key metabolic regulator in cancer. In addition, CD147 was also shown to be implicated in the angiogenesis process via the regulation of vascular endothelial growth factor (VEGF) production in tumor and stromal cells [10].

Earlier detection of cancer can greatly increase the opportunity for successful treatment. Identify new prognostic biomarkers can be useful for cancer clinical and therapeutic management [11]. An increasing number of studies have shown CD147 as a promising biomarker for predicting prognosis in many cancers [12].

## 2. CD147 Biological Functions in Cancer: Structure and Partners

A glycoprotein belonging to the immunoglobulin family and enriched on the surface of various types of tumor and stromal cells, including epithelial cells and fibroblasts [13], CD147 was initially named TCSF (tumor cell-derived collagenase stimulatory factor) and renamed Extracellular Matrix MetalloPRoteinase INducer (EMMPRIN) based on its matrix metalloproteinase (MMP) inducer function [14]. Although increasing MMPs expression in tumor cells was initially established as the major protumoral function of CD147 [14], several subsequent studies had demonstrated that its tumor-promoting role implicates other mechanisms, of which the interaction of CD147 with MCTs, leading to increased aerobic glycolysis appears to represent a major role.

**Figure 1:**
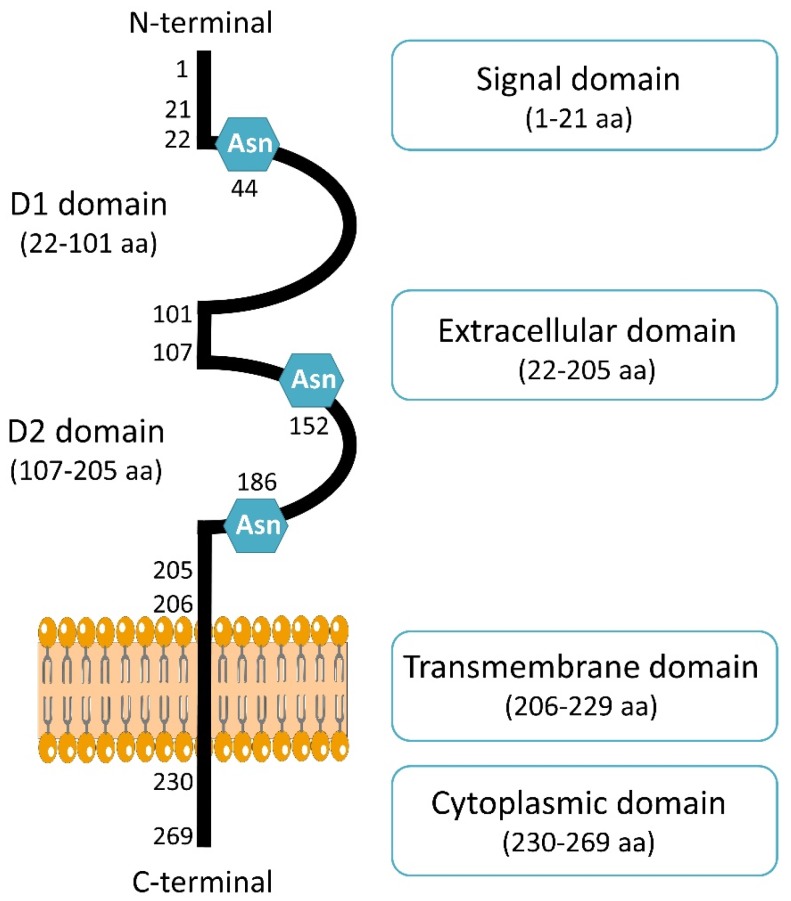
Schematic presentation of CD147 structure. CD147 consists of 269 amino acids (aa) and composed of a signal domain, an extracellular domain, a transmembrane domain and a cytoplasmic domain. CD147 contains three Asparagine (Asn) sites of glycosylation.

CD147 is coded by Basigin (*BSG*) gene located on chromosome 19p13.3 [15]. Four variants of CD147 has been encoded (Basigin 1, 2, 3 and 4) through an alternative promoter and splicing [16]. The retinal-specific CD147 (Basigin 1) containing three immunoglobulin domains [17] and isoforms Basigin 3 and 4 contain a single immunoglobulin domain [18]. The most abundant isoform and complete information is only available for Basigin 2, named CD147 in the present review. It is composed of a signal sequence of 21 amino acids, an extracellular domain of 184 amino acids with two immunoglobulin domains, a transmembrane domain of 24 amino acids, and a cytoplasmic domain of 39 amino acids [19]. Structurally, one monomer of CD147 is composed of two domains, D1 corresponding to N-terminal domain (residues 22–101) and a C-terminal domain called D2 (residues 107–205) (Figure 1). Crystal structure showed CD147 monomers can associate with each other, leading to dimer formation [20]. It was first shown that CD147 dimerization can occur in the same cell [21]. This homodimerization on the plasma membrane is in a cis-dependent manner [22]. CD147 can also interact with other cells through CD147/CD147 interaction in trans-dependent manner, which can induce intracellular signaling [23]. Moreover, a soluble form of CD147 was shown to be internalized through surface CD147 binding and enhance proliferation and migration [24]. This interaction can then induce cell surface expression of CD147 [24]. Dimerization of CD147 is crucial for MMPs induction and cell invasion in hepatocellular carcinoma though MAPK pathway [25].

Cancer-associated fibroblasts (CAFs) are the most abundant components of tumoral stroma and contribute to the malignant phenotype of cancers. In 2019, Aoki et al. demonstrated that CD147 can stimulate adjacent fibroblasts thought CD73 interaction, increasing the secretion of MMP-2 and promoting invasion and metastasis [26]. CD147 has been shown to be implicated in the transformation of normal fibroblasts to cancer-associated-fibroblast through cancer–stroma interaction and the induction of alpha smooth muscle actin (α-SMA) expression, a marker of CAFs, promoting epithelial-to-mesenchymal transition of breast cancer cells [27]. CD147 contains three asparagine (Asn) glycosylation sites in the extracellular region. The glycosylation level of CD147 is related to its different molecular weights, which have been described and classified as low and high glycosylated CD147 forms [22]. The glycosylation of CD147 was shown to be necessary for its function, since the unglycosylated forms of CD147 were not able to induce fibroblast’s MMPs production [28].

An important mechanism of action of CD147 is that it can be secreted by cells and released into the microenvironment. Two different ways by which the cell can generate these soluble forms of CD147 have been described; one involving an MT1–MMP-mediated cleavage of surface-bound CD147 [29] and a second is based on the release of microvesicules, containing full-length of CD147 molecules [30]. Recently, the interaction between ADAM12 and CD147 was shown to promote the cleavage of CD147 releasing its soluble form in the microenvironment, showing a new way to generate soluble CD147 [31]. A soluble form of CD147 was reported to be associated with tumor growth, metastasis formation and chemoresistance, and has been proposed as a clinical biomarker in breast cancer [32] and hepatocellular carcinoma [33].

CD147 is known to interact with numerous partners such as MCTs [34], caveolin-1 [35], CD98 and β1 integrin [36] to promote cell metabolism, proliferation, migration and invasion [37].

A major protumoral action of CD147 was shown to involve a metabolic modification of the tumor microenvironment through its interaction with certain MCTs (MCT-1, MCT-4) that regulate tumor glycolysis via lactic acid export. By acting as a chaperone of MCTs, CD147 facilitates their cell membrane localization and functionality and increases tumor cell aerobic glycolysis, a hallmark of metastatic cancer [38,39]. Studies by Pouyssegur‘s group, who explored the role of CD147 in MMPs induction, furthermore suggested that the previously well-established MMPs induction function of CD147 only plays a minor role in cancer progression compared to that of CD147-MCT [40]. Grandja et al. showed by a direct interaction that CD147 can regulate MCTs expression and activity in non-small lung cancer. Indeed, CD147 knockout induced a decrease of the expression and the activity of MCT-1 and MCT-4 [41]. Glycolysis and cellular activities were found significantly inhibited after CD147 silencing with RNAi technology. A study in thyroid carcinoma cells showing a close association between CD147 and MCTs confirmed the dominant role of CD147 in glycolysis [42]. In multiple myeloma cells, CD147 was showed to act as a chaperone of MCT-1, thus promoting tumor growth within an acidic microenvironment [43]. These results reinforce the importance of the interaction between CD147 and MCTs as a major mechanism in tumor progression.

Other cell surface partners of CD147 have been described. Caveolin-1 was shown to interact with CD147 and this had a negative effect on the MMP-inducing function of CD147 [44]. CD98 and CD147 form a complex with MCTs and play a critical role in the energy metabolism of the cell [45]. Cell motility was shown to be regulated by CD147/integrin interaction through FAK-STAT3 pathway [46]. CD147 overexpression also directly contributes to tumor angiogenesis by simulating VEGF production via the PI3K/AKT pathway [47].

**Figure 2:**
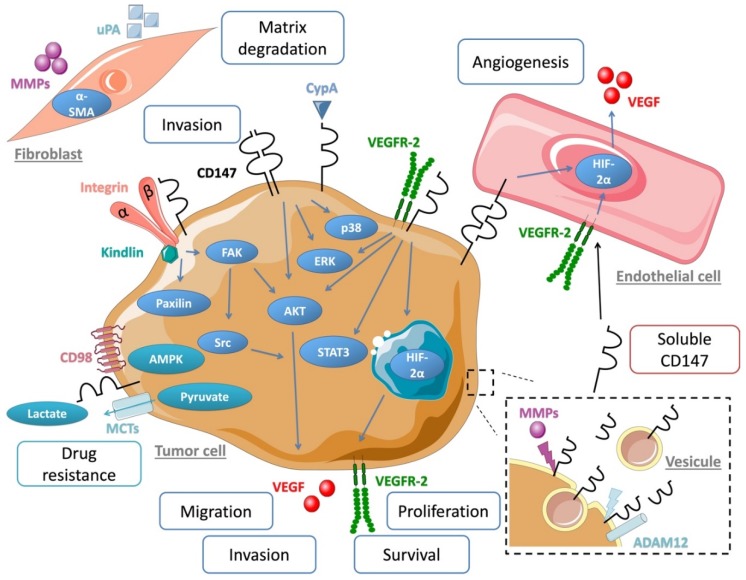
Schematic overview of CD147 associated partner, molecular pathway and microenvironment interaction involved in cancer progression.

The ABC transporter G2 (ABCG2) is known to be involved in drug resistance [48,49], thanks to its role in drug transport and efflux [50]. CD147 can regulate ABCG2 cellular location and dimerization [51], leading to cancer cell chemoresistance [52]. CD147 was also shown to be a signaling receptor for cyclophilin A (CypA), a cytosolic protein secreted in response to inflammatory stimulations. CypA is overexpressed in cancer and leads to malignant transformation and metastasis via ERK1/2 signaling [53]. CD147 interaction with CypA was shown to induce cancer cell proliferation [54] and regulates cell immunity and inflammation (Figure 2) [55].

## 3. CD147 Regulates Cancer Cell Invasion and Metastasis

Tumor invasion depends on a complex mechanism involving cell adhesion, migration and matrix degradation. In addition, the alteration in the surrounding microenvironment is known to further promote invasion by cooperating with the tumor cell and supporting its survival, proliferation and production of proteinases essential for degrading the extracellular matrix, mainly the MMPs and the serine proteases (uPA, plasmin). CD147 enriched on the surface of tumor cells, was shown to be an important factor in tumor stroma interactions, as it stimulates neighboring stromal to increase their synthesis of several MMPs [14,56]. The extracellular N-terminal region of CD147 was shown to be crucial for the MMPs induction [14,57]. Although MMPs induction may not represent the major tumor-promoting function as was previously thought, by degrading the surrounding stroma, MMPs are important in regulating cell growth, migration and invasion. CD147 was shown to stimulate the production of MMP-1, MMP-2, MMP-3 in melanoma [58] and MMP-9 in breast cancer [59] but had no effect on the specific tissue inhibitors of MMPs, TIMP-1 and TIMP-2 [60]. Following activation, MMP-9 lead to degradation of type IV collagen and promoted tumor cell invasion and metastasis in breast cancer [61]. CD147 and MMP-11 were overexpressed in colorectal cancer and showed a colocalization between both proteins. In addition to its role in invasion and metastasis, MMP-11 was shown to confer resistance to anoikis, a process of apoptosis induction in absence of attachment to the extracellular matrix [62], a process frequently observed in cancer cells [63]. In this context, CD147 has also been described as a suppressor of anoikis by contributing to the malignant phenotype in breast cancer [64] and hepatocellular carcinoma [65].

Our group has previously shown that CD147 also induces expression of the urokinase plasminogen activator system, uPA/uPAR/PAI-1, in melanoma, breast and ovary tumor cells, further increasing its proteolytic and invasion potential in vitro and in vivo [66]. Indeed, increasing CD147 expression experimentally in human breast cancer cells greatly enhanced tumor growth and metastases in nude mice and was associated with an increase in MMPs and urokinase production in the tumors [66,67]. By transcriptome analysis of individual tumor cells isolated from bone marrow of cancer patients and comparative genomic hybridization technique, CD147 was shown to be the most frequently expressed protein in primary tumors and in micrometastatic cells [68], suggesting a central role in tumor progression and early metastasis.

Furthermore, the invasive proprieties of breast cancer cells are modulated by the interaction of CD147 and hyaluronan-CD44 though the activation of EGFR–RAS–ERK pathway. Our previous demonstration that CD147 expression in epithelial mammary tumor cells is increased by the EGF/EGFR system [69] suggests that the activation of EGFR signaling may account, at least partially, to the increased expression of CD147 observed in most carcinomas and to the therapeutic potential of EGFR inhibitors. More recently, the role of CD147 in promoting tumor progression and metastasis was also reported in head and neck squamous carcinoma through the NF-κB pathway [70].

## 4. CD147 Regulates Tumors Cells Adhesion

Integrins are major cell surface adhesive receptors composed of α- and β-chain heterocomplexes that mediate cell matrix adhesion and migration, playing an important role in the invasive process of tumor cells. Integrins control ECM remodeling by the regulation of the localization and activity of proteases [71]. Several studies have described CD147–integrin interactions, which can regulate adhesion and migration in cancer cells. CD147 forms complexes with α3β1 and α6β1 integrins at cell–cell contact [72], an interaction that promotes cancer invasiveness by inducing MMP synthesis via a focal adhesion kinase (FAK)-PI3K signaling pathway [73,74].

In human hepatoma cells, overexpression of CD147 was shown to promote invasion and metastasis via α3β1 FAK-paxillin and FAK-PI3K-Ca2+ pathways [75]. Furthermore, α3β1 and CD147 co-localize on human 7721 hepatoma cells [76]. In oral cancer cells, the interaction of CD147 with β6 integrin was shown to cooperate with Fyn, a Src family kinase, in modulating MT1-MMP activity [77]. CD147 and MT1-MMP were shown to be in close proximity within invadopodia-like structures [78]. CD147 knockdown decreased the ability of prostate cancer cells to form filopodia and promote cell adhesion, which demonstrate the capacity of CD147 to regulated cytoskeleton rearrangement [79].

Our study, using a transcriptomic approach aimed at identifying CD147 regulated partners involved in cell adhesion and invasion, revealed Kindlin-3 (an integrin partner), to be inversely regulated by CD147 [80]. Kindlin-3 (also called FERMT3 or Mig2B), is an integrin-interacting focal adhesion protein that mediates integrin activation [81]. Kindlin-3 has been implicated in β2 integrin activation in leukocytes and was shown to promote their adhesion and endothelial transmigration [82,83]. Recently we have shown that Kindlin-3 acts as a tumor suppressor in solid tumors *in vitro* and *in vivo* [84]. Functional studies allowed us to demonstrate in melanoma models, that Kindlin-3 is involved in CD147 regulation of β1 integrin-mediated adhesion [80].

## 5. CD147 Promotes Tumor Angiogenesis

CD147 was also shown to be involved in tumor angiogenesis, a key component of the tumor microenvironment. MMP expression induced by CD147 in both tumor and stromal compartments in turn releases biologically active angiogenic growth factors from matrix-bound complexes. In 2005, Tang et al. showed that CD147 stimulates tumor angiogenesis by increasing VEGF and MMP expression levels in both tumor and stromal compartments [85]. Our studies have shown that tumoral CD147 increased the production by endothelial cells of VEGF soluble isoforms (particularly the most angiogenic isoforms) and of its main receptor VEGFR-2 through the transcription factor HIF-2α, both in vitro and in experimental tumor models in vivo. Moreover, CD147 promotes capillary-like formation, migration and cell survival through VEGFR-2 and its ligand VEGF [10]. We have also shown that this regulation of VEGF/VEGFR-2 by CD147 is not limited to endothelial cells and can be also observed in melanoma tumor cells leading to an increase of their malignant properties [86]. Further studies allowed us to identify a unique mechanism of action of CD147, showing that its direct interaction with VEGFR-2 on the plasma membrane is required for VEGF induced VEGFR-2 activation. This VEGFR-2 co-receptor role of CD147 has been studied using computational docking analyses and mutagenesis and led to the identification of a molecular binding site in the extracellular domain of CD147 located close to the cell membrane and containing the amino acids 195/199. The overexpression of CD147 in cancer is able to further potentiate VEGFR-2 activation, suggesting that a combinatory therapy of an antiangiogenic drug together with an inhibitor of CD147/VEGFR-2 interaction may have a greater impact on inhibiting angiogenesis and malignancy [87].

CD147 is also implicated in lactate efflux, via its cotransporter monocarboxylate transporter 4. Indeed, MCT4 regulates CD147 maturation and trafficking to the plasma membrane in breast cancer cells [88]. Accumulation of lactic acid in the ECM is also known to promote angiogenesis via the increase of VEGF/VEGFR-2 synthesis by tumor and endothelial cells, which reinforce the role of CD147 in the regulation of tumor angiogenesis.

## 6. CD147 Therapeutic Targeting Strategies

Drug resistance is a major issue in cancer therapy that promotes treatment failure and patients’ relapse. A big challenge is the identification of those patients who will develop a therapy resistance and the setup of more effective alternative therapeutic strategies. A number of studies brought several levels of evidence that converge to a role of CD147 in drug resistance. CD147/CD98hc complex (a high glycosylated chain linked with a low glycosylated chain highly expressed on human tumor cells) is found overexpressed in cisplatin-resistant cancer cell lines [89]. CD147 expression increased chemotherapeutic drug resistance (Doxorubicin, BCNU, Taxol and Vincristine) via hyaluronan [90] and CD44 interaction, including receptor tyrosine kinase, ABS transporter and MCTs activities facilitating drug efflux with resistance to cisplatin and methotrexate in head and neck cancer [91], to cisplatin in lung cancer [92], and to vincristine in lymphoma [93]. Cooperation between CD147 and the lymphatic vessel endothelial hyaluronan receptor-1 (LYVE-1) was also described in the regulation of chemoresistance in lymphoma through upregulation of the drug transporter/ABCG2 (BCRP, the breast cancer resistance protein) [94]. Another interaction between CD147 and ABCG2 has been involved in breast cancer chemoresistance. Indeed, CD147 can bind to ABCG2 to form a complex that maintains it stability [51]. In the light of these works, CD147-targeted therapy could be a potential approach to bypass such drug resistance.

An antibody (MEM-M6/1) directed against CD147 and MCT-1 interaction was shown to induce necrosis-like cell death in colon cancer cells and melanoma cells. Moreover, MEM-M6/1 inhibited the lactate release [95]. More recently, a drug-screening assay identified Acriflavine (ACF), a small molecule responsible for the inhibition of CD147 and MCT-4 interaction and as a result of glioblastoma tumor growth and angiogenesis [96].

CD147 blockade with a specific antibody strategy inhibited secretion of MMP-9 and VEGF in a dose-dependent manner. Indeed, the antibody (161-Ab) reduced *in vivo* tumor growth even when used in well-established tumors and decreased development of metastasis. Interestingly, the 161-Ab antibody showed a better effect at the lower doses than higher ones [97]. In pancreatic cancer, antibody 059-053 directed against CD147, combined with gemcitabine, suppressed tumor growth and decreased tumor cell survival in xenograft mouse models [98]. Another anti-CD147 monoclonal antibody (CNTO3899) induced tumor cell apoptosis with an increase of caspase 3 and caspase 8 in human head and neck tumor tissues [99]. CD147 and MMP-9 expression led to EGFR expression and contributed to tumor progression [69]. Anti-CD147 therapy led to synergy in combination with inhibition of EGFR and reduced head and neck squamous cell carcinoma (HNSCC) cell proliferation and migration [100]. In 2006, Wang et al. produced four antibodies against CD147 and observed two different effects in hepatocellular carcinoma. The antibodies 1B3 and 3B3 were able to inhibit MMP-2 secretion and cell invasion while the two others (HAb18Gedomab1 and HAb18Gedomab2) showed opposite effects. This discrepancy was explained by the fact that there are two different epitopes in the extracellular domain that appear to differently control MMPs production, one an agonist, and the other an antagonist [101]. Further studies identified the residues ^22^AAGTVFTTVEDLGSKILLTCSLNDSATEV^50^ critical for MMPs induction [102].

To target CD147, a therapeutically agent Licartin (generic name (I^131^) metuximab injection) was developed as an anti-CD147 monoclonal antibody HAb18 [103] conjugated to the radioisotope I^131^. In phase I/II trials, the Licartin was shown to be safe and was officially approved by the China State Food and Drug Administration (SFDA, Registration No. S20050039) [104]. A randomized trial showed that Licartin can prevent relapse after liver transplantation in hepatocellular carcinoma [105]. More recently, HAb18 was shown to sensitized pancreatic cancer cells to chemoradiotherapy (gemcitabine and genfitinib) through suppressing STAT3 pathway [106]. Although Licartin has been efficient against liver cancer, its radioactive I^131^ component would limit its application.

Different forms of the anti-CD147 HAb18 were developed, a chimeric antibody, cHAb18, containing variable heavy and light chains of the HAb18 antibody and constant regions of human IgG1γ1, modulated cytoskeleton rearrangement via FAK-PI3K-AKT signaling pathway and inhibited invasion and metastasis in hepatocellular carcinoma [74]. HcHAb18 antibody conjugates with a cytotoxic drug that is a maytansinoid derivative (DM1) to promote antitumor activity in lung cancer [107].

Beside the anti-CD147 antibody strategy, a small molecule (AC-73) inhibitor of CD147 dimerization has been shown to decrease MMP-2 production via CD147-ERK-STAT3-MMP-2 pathway in hepatocellular carcinoma. AC-73 permitted the loss of invasiveness and decrease motility, resulting in the inhibition of metastasis formation [108]. The overexpression of CD147 in acute myeloid leukemia cells was shown to promote cell proliferation. AC-73 treatment led to the inhibition of leukemic cells (NB4 and NB4-R4) proliferation via the inactivation of the ERK-STAT3 pathway, as well as autophagy induction. Moreover, AC-73 was able to enhance the sensitivity to chemotherapeutic treatment and allowed a decrease in chemotherapy dosing (arabinosylcytosine and arsenic trioxide) [109].

Sato et al. reported that high expression of CD147 in renal cell carcinoma (RCC) is associated with resistance to the anti-tyrosine kinase receptor inhibitor, sunitinib, suggesting therefore that CD147 blockage in association to tyrosine kinase receptors inhibitors could restore sensitivity to such targeted therapy in RCC [110].

## 7. CD147 as a Prognostic Biomarker

Numerous studies underscored the implication of CD147 in tumor progression, suggesting its role in tumor prognosis. CD147 expression was found to be increased in more than 20 types of cancers from different organs and its overexpression has been shown to be constantly associated with poor tumor outcome (Table 1), and hence to represent a strong prognostic impact [111] including overall survival and progression free survival.

In our studies, using a large series of 196 cutaneous melanomas including primary and metastatic melanomas, we showed that high CD147 expression, assessed by immunohistochemistry, was significantly associated with the metastatic potential and with a reduced overall survival in primary melanoma patients. CD147 expression level was correlated with Clark level, ulceration status and more importantly with the Breslow index, which was associated with prognosis. In multivariate analysis, CD147 remains an independent prognostic biomarker and emerged as an important factor in the aggressive behavior of melanoma [112]. The prognostic value of CD147 expression was also shown in bone cancer. Higher expression was found in clinical stage III compared to stage I/II and was associated with aggressiveness of tumor bone cells [113]. A significant association between the expression of CD147 protein and oral squamous carcinoma (OSCC) stage was reported in a cohort of 100 tissue blocks [114]. Our previous studies in OSCC tumors demonstrated increased expression of CD147 compared to dysplastic lesions. A similar expression pattern was observed in the precancerous and invasive corresponding oral tumoral cell lines [115]. Similarly, Vigneswaren et al. reported CD147 expression in dysplastic leukoplakias, which was correlated with the degree of dysplasia. This expression was greatly increased with progression to primary and metastatic OSCC. These studies imply that overexpression of CD147 can occur at a very early stage of oral carcinogenesis and may contribute to OSCC tumorigenesis [116].

Other studies have also reported a correlation between CD147 levels and prognosis. In a cohort of 150 primary lung adenocarcinomas, Sienel et al. showed that membranous staining of CD147 was associated with shortened survival independently of MMP-2 and MMP-9 expression. This study suggests, therefore, that the prognostic predictor role of CD147 could be unrelated to its function as inducer of MMPs [117]. More recently in 2017, Liu et al. reported that CD147 overexpression within the tumor lesions of non-small lung cancer was associated with lymph node metastasis and progression to more advanced stages. In addition, high CD147 levels in the serums of those patients was also associated with cancer progression [118].

In a series of 2222 human breast cancer tissues, CD147 expression level assessed by immunohistochemistry was associated with tumor grade and also with tumor size. CD147 expression was detected in most micrometastatic tumor cells. More importantly, high CD147 expression level was a strong and independent predictor of shorter survival [119]. Recently in a large series of 1174 breast cancer subtypes, CD147 expression was correlated with high tumor grade, presence of necrosis and high Ki67 expression [120]. Furthermore, CD147 expression was associated with poor survival in patients with triple-negative breast cancer subtype treated with chemotherapy [120]. In ovarian cancer, CD147 was detected in primary tumors and in metastases sites and its expression in the primary tumors was correlated with poor survival [121]. Increased expression of CD147 was also reported in glioblastoma, where it was much increased compared to normal brain tissues and its overexpression was associated with poor rates of survival of patients overall [122].

In a cohort of 53 renal cancers, combination of CD147 and VEGF expression was found to predict tumor prognosis. VEGF expression correlated with CD147 expression and promoted tumor progression [123]. Additionally, CD147 overexpression seemed to appear at the later, more aggressive stages of renal cancer [124]. In a cohort of 120 primary prostate cancers, the histological grade, clinical stage, nodal involvement and progression was associate with high CD147 expression [125]. Furthermore, Zhong et al. showed the CD147 overexpression as a significant predictor for metastasis and survival in prostate cancer [126]. In advanced or metastatic bladder cancer, CD147 expression predicted the response to cisplatin-containing chemotherapy. Negative CD147 expression was associated with a better response and survival to cisplatin-containing chemotherapy treatment in a cohort of 124 bladder cancer patients [127]. Moreover, this finding was also validated in the neoadjuvant setting of bladder cancer. Patients with positive CD147 expression showed an efficacy disadvantage with neoadjuvant therapy compared to patients with a negative expression of CD147 [128].

The prognosis of gastrointestinal cancers is also linked to CD147 expression. Indeed, a high level of CD147 has been shown to correlate with the progression of gastric carcinoma and elevated CD147 expression was suggested to enhance growth, with angiogenesis allowing more ability to invade into vessels [129]. A meta-analysis of 17 studies highlighted the implication of CD147 expression in the risk of esophagus cancer. Elevated expression induced invasion, metastasis and poor survival and was suggested to be important for estimating prognosis [130]. The independent worse prognostic value of CD147 expression also reported in colorectal cancers. A study evaluating a series of 285 patients with colorectal cancers have shown that CD147 increased expression was correlated with poor survival although the increase was not correlated with the clinicopathologic parameters such as stage and metastasis in this study [131].

In acute myeloid leukemia, the analysis of bone marrow biopsies from a cohort of 62 patients revealed a significant association between expression of CD147 and VEGF. Indeed, high levels of co-expression has been suggested to induce an unfavorable prognosis by supporting leukemic cell growth [132].

Together, these studies showing the association between CD147 overexpression and disease outcome in most types of cancers strengthen the importance of the role of CD147 in tumor progression and validate its potential value as a strong prognostic biomarker.

## 8. Conclusions

Tumor management strategies include earlier cancer detection and understanding of the mechanisms that drive malignancy through mediating crucial processes such as invasion, metastasis, metabolism, angiogenesis and chemoresistance. The tumor microenvironment represents a wealthy source for potential prognostic markers and therapeutics targets. CD147 overexpression is a worse prognostic factor of numerous types of cancers, including solid cancers as well as hematological malignancies, which is strongly based on its functions enhancing tumor cell malignant properties. Altogether, these data underscore CD147 as a promising biomarker in cancer and also, through its successful targeting, as a potential antitumor target paving the way to new anti-CD147 therapeutic strategies.

## Figures and Tables

**Table 1:** Studies reporting CD147 as a prognostic biomarker.

Reference	First Author	Year	Type of Cancer	Sample Size	Comments
[113]	Si et al.	2003	Bone cancer	19	CD147 expression associated with progression and aggressiveness
[121]	Davidson et al.	2003	Ovarian cancer	130	CD147 is expressed in all sites (effusions, primary tumor and solid metastases) and predict poor prognosis
[119]	Reimers et al.	2004	Breast cancer	2222	CD147 expression in primary tumor predicts a poor prognosis
[116]	Vigneswaran et al.	2006	Oral cancer	140	CD147 overexpress in advanced primary and metastatic tumors
[129]	Zheng et al.	2006	Gastric carcinoma	319	CD147 expression linked to tumor size
[127]	Als et al.	2007	Bladder cancer	124	CD147 expression predict response to Cisplatin-containing chemotherapy
[117]	Sienel et al.	2008	Lung cancer	150	Membrane localization of CD147 was associated with poor survival independently of MMP-2 and MMP-9
[125]	Madigan et al.	2008	Prostate cancer	120	Higher expression of CD147 in high grades
[123]	Liang et al.	2009	Renal cancer	53	CD147 expression correlated with VEGF expression and played a role in progression
[132]	Fu et al.	2010	Acute myeloid leukemia	62	Co-expression of CD147 and VEGF promote unfavorable prognosis
[131]	Stenzinger et al.	2011	Colorectal cancer	285	CD147 expression decreased survival
[115]	Lescaille et al.	2012	Oral cancer	20	CD147 expression increased with invasive stage
[126]	Zhong et al.	2012	Prostate cancer	240	CD147 expression can serve as a significant marker for progression
[122]	Yang et al.	2013	Glioblastoma	206	High CD147 expression mediated poor overall survival
[124]	Rabien et al.	2013	Renal cancer	395	CD147 expression increased only with progression
[128]	Hemdan et al.	2015	Bladder cancer	250	Strong expression of CD147 promoted worse response to neoadjuvant chemotherapy
[112]	Caudron et al.	2016	Melanoma	196	High CD147 expression associated with metastatic potential and short survival
[118]	Liu et al.	2017	Lung cancer	72	High CD147 in serum-mediated metastasis and advanced stage
[130]	Li et al.	2017	Esophagus cancer	17 studies (1140 samples)	Worse survival and poor prognosis with CD147 strong expression
[114]	Arora et al.	2018	Oral squamous cell carcinoma	100	CD147 intensity associated with different grades
[120]	Liu et al.	2018	Breast cancer	1174	CD147 expression mediated survival in chemotherapy-treated patients
